# Supplementary material for: On the nature of hydrogen bonding in the H2S dimer
Source: Nat Commun. 2024 Nov 5;15:9540. doi: 10.1038/s41467-024-53444-6 (PMC11538508; doi:10.1038/s41467-024-53444-6)
Supplement: Supplementary file 1 — Supplementary Information [file 41467_2024_53444_MOESM1_ESM.pdf]

Supplementary Information to On the nature of hydrogen bonding in the H_2S dimer

Svenja Jäger,[†] Jai Khatri,[†] Philipp Meyer,[†] Stefan Henkel,[†] Gerhard Schwaab,[†] Apurba Nandi,[*,‡] Priyanka Pandey,[‡] Kayleigh R. Barlow,[¶] Morgan A. Perkins,[¶] Gregory S. Tschumper,[¶] Joel M. Bowman,[*,‡] Ad van der Avoird,[*,§] and Martina Havenith[*,†]

†*Department of Physical Chemistry II, Ruhr University Bochum, 44801 Bochum, Germany*

‡*Department of Chemistry and Cherry L. Emerson Center for Scientific Computation, Emory University, Atlanta, Georgia 30322, U.S.A.*

¶*Department of Chemistry and Biochemistry, University of Mississippi, University, Mississippi 38677-1848, U.S.A.*

§*Theoretical Chemistry*
Institute for Molecules and Materials, Radboud University
Heyendaalseweg 135, 6525 AJ Nijmegen, The Netherlands

E-mail: apurba.nandi@emory.edu; jmbowma@emory.edu; A.vanderAvoird@theochem.ru.nl; martina.havenith@rub.de

Svenja Jäger and Jai Khatri contributed equally

Suppl. Note 1 Potential and dipole moment surfaces

Δ-Machine learned potential energy surface

Our approach is to construct a high-level, typically CCSD(T), potential energy surface (PES) starting from a lower level MP2 or DFT one using a correction which is a fit to a small number of high-level *ab initio* energies. Explicitly, the corrected high-level PES, denoted $V_{LL\to CC}$, is given by

$$V_{LL\to CC} = V_{LL} + \Delta V_{CC-LL}, \tag{1}$$

where V_{LL} is the lower-level PES and ΔV_{CC-LL} is the correction PES based on high-level coupled-cluster energies.[1] It has been observed that the difference between CCSD(T) and MP2 energies, ΔV_{CC-LL}, is not as strongly varying as V_{LL} with respect to the nuclear configurations and therefore just a small number of high-level electronic energies are adequate to fit the correction PES. In the present application to H_2S-dimer, first we computed a total of 9149 MP2/ha(T+d)Z electronic energies and their corresponding gradients to fit the V_{LL} PES and then a subset of this data in the size of 2319 CCSD(T)-F12b/haQZ-F12 (defined in the last paragraph on the next page) electronic energies were computed to correct the V_{LL} PES.

Here we employ the permutationally invariant polynomial (PIP) approach to fit both the V_{LL} and ΔV_{CC-LL} PESs. The theory of permutationally invariant polynomials is well established and has been presented in several review articles.[2–6] In terms of a PIP basis, the potential energy, V, can be written in compact form as

$$V(\boldsymbol{x}) = \sum_{\alpha=1}^{n_p} c_\alpha p_\alpha(\boldsymbol{x}), \tag{2}$$

where c_α are coefficients, p_α are PIPs, n_p is the total number of polynomials for a given maximum polynomial order and $\boldsymbol{x}$ are Morse variables. For example, x_{ij} is given by $\exp(-r_{ij}/\lambda)$, where r_{ij} is the internuclear distance between atoms i and j. The range (hyper)parameter,

λ, was chosen to be 2 bohr. The coefficients c_α are obtained using standard least squares methods for a large data set of electronic energies (and for large molecules' gradients as well) at scattered geometries.

The low-level PES, V_{LL}, is developed using the MP2/ha(T+d)Z (aug-cc-pV(T+d)Z basis for S and cc-pVTZ for H) level of theory. The simultaneous energy-gradient fitting has been employed to fit the MP2 PES. The dataset of energies and corresponding gradients were generated from *ab initio* molecular dynamics (AIMD) simulations at several total energies by using microcanonical sampling (NVE). Initial conditions were chosen to obtain a wide coverage of the configuration space for each isomer. The main purpose of this PES is to describe the bound states of the weakly bound H_2S dimer. The dataset of energies and gradients was obtained using direct dynamics, with the following sets of initial conditions. In one case, a batch of trajectories was run at each of the three equilibrium structures found in Ref. 7 with kinetic energies of 250, 500, 1000, 3000, and 5000 cm^{-1}. Another batch of trajectories was run at the global minimum with the kinetic energies of 10000, 20000, and 30000 cm^{-1}. However, these high energetic trajectories blew up after some point as it is a weakly bound system. Thus, high energies in the repulsive short-range region, needed for the rigid-monomer calculations, are obtained from cuts in R with five internal angles varied. These calculations were done at the MP2/ha(T+d)Z level of theory, using the Molpro quantum chemistry package.[8] The final data set consists of 9149 energies and corresponding 164 682 gradients for a total size of 173 831.

To develop the correction PES, ΔV_{CC-LL}, a data set of 2319 geometries is sparsely selected from the 9149 MP2 dataset by selecting every 7th geometry. Then explicitly correlated CCSD(T)-F12b[9–15] single-point energy computations are performed on these 2319 geometries, with no scaling applied to the triples energy contribution. The basis set used along with this method is Dunning's quadruple-ζ atomic orbital (AO) correlation consistent basis set, optimized for use with explicitly correlated methods and including diffuse functions on sulfur atoms (i.e., cc-pVQZ-F12[16,17] for H and aug-cc-pVQZ-F12[17,18] for S), denoted haQZ-F12.

The default resolution of the identity and density fitting auxiliary basis sets[19–21] defined in Molpro[22,23] are employed for all of the explicitly correlated computations.

We add the correction $\Delta V_{\text{CC-LL}}$ to the low-level MP2 PES, V_{LL} to obtain the CCSD(T) energies. The root mean square (RMS) difference between the $V_{LL\to CC}$ and direct CCSD(T) energies is 60 cm^{-1}. We perform geometry optimizations and normal-mode frequency calculations at the three low-lying minima and their two isomerization saddle point geometries to examine the fidelity of the new $V_{LL\to CC}$ PES.

The relative energies of these minima are given in Table 1 along with the previously reported numbers. Note that both MP2 and CCSD(T) energies for all these stationary points are very small, suggesting that these geometries are sampled by the nuclear motion in the ground vibrational state. This is verified in full-dimensional diffusion Monte Carlo calculations, described below.

Suppl. Table 1: Comparison of relative electronic energies (in cm^{-1}).

	V_{LL}		$V_{LL\to CC}$	
Minimum	PES	Direct MP2[a]	PES	CCSD(T)[a]
I	0	0	0	0
II	50	16	43	23
III	58	28	56	28
TS1	52	36	46	32
TS2	66	53	51	45

[a] From Ref. 7

Next, to examine the vibrational frequency predictions of the PES, we performed normal mode analyses for all three low-lying conformers and their isomerization saddle points. The comparison of harmonic mode frequencies for Minimum I with their corresponding *ab initio* ones is shown in Table 2. The agreement with the reported CCSD(T) benchmark frequencies[7] is overall very good; the maximum errors are 28, 43, and 24 cm^{-1} for some low-frequency modes of conformers I, II, and III, respectively, but most of the frequencies are within a few cm^{-1} of the *ab initio* ones and the mean absolute errors (MAE) are only 10, 15, and 8 cm^{-1}.

The two isomerization saddle point geometries are confirmed by obtaining one imaginary frequency. Results for Minimima II and III are very similar and so not shown here.

Suppl. Table 2: Comparison of harmonic frequencies (in cm^{-1}) for Minimum I.

	V_{LL}		$V_{LL\to CC}$	
Mode	PES	MP2[a]	PES	CCSD(T)[a]
1	30	36	29	33
2	70	61	65	56
3	86	70	80	67
4	92	78	84	74
5	166	157	161	147
6	241	276	228	256
7	1223	1212	1215	1208
8	1233	1218	1227	1214
9	2732	2740	2689	2699
10	2775	2778	2712	2720
11	2788	2792	2735	2732
12	2789	2797	2735	2736

[a] From Ref. 7

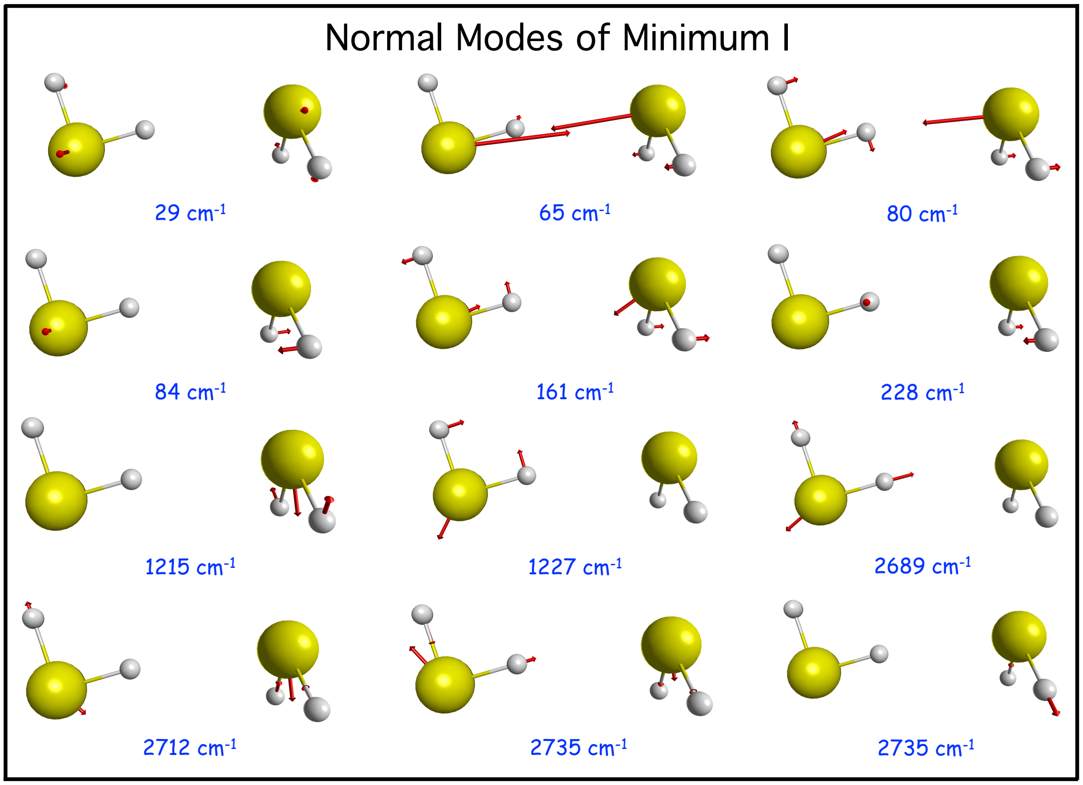

Suppl. Fig. 1: Mass-weighted normal mode eigenvectors and corresponding frequencies for Minimum I.

Dipole moment surface

As previously, we express the dipole moment surface (DMS) as[2,24]

$$\mathbf{d} = \sum_{i=1}^{6} \mathbf{r}_i q_i, \tag{3}$$

where the q_i are the machine-learned charges of each atom center with coordinates $\mathbf{r}_i$. In the present case there are two unique q_i, one for S and the other for H. These machine-learned charges are expressed as permutationally invariant scalars which are functions of all Morse variables, much like the potential. However, the invariance symmetries are different. For example, the q_i for the H atoms are invariant with respect to the interchange of the two S atoms. Similarly the q_i for the S atoms are invariant with respect to all permutations of H atoms.

The dipole moment was obtained at each dimer configuration of 9149 geometries at the efficient MP2/ha(T+D)Z level of theory. The fit was done (each component of the dipole vector) with the permutational symmetry group $S_4 \otimes S_2$ (describing the identity of the four hydrogen atoms and the two sulfur atoms) and a maximum polynomial order of 5. This leads to 1810 linear expansion coefficients, which are determined by solving a linear least-squares problem. The fitting RMS errors for the X, Y, Z components are respectively, 0.018, 0.021, 0.021 Debye (D) and the overall RMS error is 0.020 D.

Suppl. Note 2 Vibration-rotation-tunneling states and far-IR spectrum

Theory

The calculations of the vibration-rotation-tunneling (VRT) states of the H_2S dimer are based on the rigid-monomer approximation, justified by the fact that the intramolecular vibrations

have much higher frequencies than the intermolecular modes. They use a six-dimensional (6D) potential surface which depends on six Jacobi coordinates: the length R of the vector $\boldsymbol{R}$ that points from the center of mass of monomer A to that of monomer B, and the Euler angles $\boldsymbol{\omega}_{\mathrm{X}} = (\alpha_{\mathrm{X}}, \beta_{\mathrm{X}}, \gamma_{\mathrm{X}})$ that define the orientations of the monomers X = A and B in a body-fixed (BF) frame with its z-axis along $\boldsymbol{R}$. Actually the 6D potential depends only on the dihedral angle $\alpha = \alpha_{\mathrm{B}} - \alpha_{\mathrm{A}}$. Also the VRT states depend on these angles, and in addition on the angles (Θ, Φ) which define the orientation of $\boldsymbol{R}$ in a space-fixed (SF) frame. The 6D rigid-monomer potential is obtained from the full 12D potential surface described in the preceding section by adopting a C_{2v} symmetric geometry for both H_2S monomers with SH bond length 1.3384 Å and HSH angle 92.5°. This geometry is nearly the same as that of the acceptor, which is the least perturbed monomer, at the dimer equilibrium geometry in the 12D potential.

The 6D large-amplitude intermolecular vibrations and tunneling motions of the H_2S dimer for rotational states with total angular momenta $J \leq 2$ are calculated with two different methods. Both are based on the Hamiltonian in a two-angle embedded body-fixed (BF) frame defined in Refs. 25,26 and they use the same analytic BF basis defined in Eq. (16) of Ref. 26. The first method is the split Wigner pseudospectral approach[27] that employs a grid basis in all six intermolecular coordinates in combination with the analytic basis, applies the iterative Lanczos method[28] to obtain the eigenvalues and eigenfunctions of the Hamiltonian, and includes very efficient transformations of the Lanczos vectors from the grid basis to the analytic basis, and vice versa. The second method uses only the analytic angular basis, expands the 6D potential in angular expansion functions given in Eq. (9) of Ref. 26 with expansion coefficients obtained as in Eq. (11), and calculates the angular matrix elements of the potential analytically with Eq. (18) of this reference. This second method, which applies the Davidson algorithm[29] to iteratively obtain the eigenvalues and eigenvectors, is explained in detail in Ref. 30.

Also the far-infrared spectrum was calculated with two different methods. The first

method uses recursive residue generation[31,32] to directly get the transition dipole moments as the projections of eigenstates multiplied with the dipole function onto the initial Lanczos vectors without actually computing all final eigenstates, which can be efficiently done in the grid basis of the split Wigner pseudospectral approach. The second method expands not only the potential but also the dipole moment function in angular basis functions, see Eqs. (35) to (38) in Ref. 26, and analytically calculates the transition dipole moment over the initial and final states with the aid of Eqs. (A6) and (A11) in Ref. 33. The dipole moment function in BF intermolecular coordinates is obtained from the 12D dipole moment surface described above in the same way as the rigid monomer intermolecular potential is derived from the 12D potential surface, with the same fixed H_2S monomer geometry.

The symmetry group of the H_2S dimer in its equilibrium geometry is the point group C_s. But the dimer has eight equivalent equilibrium structures with rather low energy barriers between the corresponding global minima in the potential, so the symmetry used in our calculations is the permutation-inversion (PI) or molecular symmetry[34] group G_{16}. This group has 10 irreducible representations (irreps) listed in Table 5 of the main paper. The use of these G_{16} irrep symbols is rather general, but their definition varies, so we state explicitly in this table which convention we use. We note here that for the H_2S monomer, just as for H_2O, one denotes rotational states with the asymmetric rotor quantum numbers $j_{k_a k_c}$. The most abundant sulfur isotope ^{32}S has nuclear spin 0, just as ^{16}O. States that are even under the permutation P_{12} that interchanges the two H atoms have even $k_a + k_c$, are called para-H_2S (pH_2S), and have total nuclear spin 0 (multiplicity 1), and states odd under P_{12} have odd $k_a + k_c$, are called ortho-H_2S (oH_2S), and have total nuclear spin 1 (multiplicity 3). These monomer nuclear spin weights, combined with the permutation P_{AB} that interchanges the monomers A and B, cause the H_2S dimer states with different irreps to have the nuclear spin multiplicities given in Table 5 of the main paper. The symbol E^* denotes the inversion operation. In both methods to calculate the VRT states the calculations were performed separately for each irrep with a symmetry-adapted basis, as explained in Refs. 27,30.

Technical details

In the VRT calculations with the split Wigner pseudospectral method we used an analytic angular basis with j_A and j_B values ranging from 0 to 10, with k_A and k_B values ranging from -10 to 10. The corresponding angular grid was a direct product grid built with 13 Legendre quadrature angles for β_A and β_B in the range from 0 to π, and equidistant Fourier grids for $\gamma_\mathrm{A}, \gamma_\mathrm{B}$, and α each containing 24 angles. With the use of symmetry the range of the latter angles could be reduced from 0 to π. The basis for R contained 38 sine type basis functions and the radial grid consisted of 41 equidistant points ranging from 3 to 6.7 Å.

The second method based on the analytic basis only is less efficient than the pseudospectral method and the angular basis had maximum $j_\mathrm{A}, j_\mathrm{B}$ and $|k_\mathrm{A}|$, $|k_\mathrm{B}|$ values of 8. For the radial coordinate R we used an equidistant 96-point grid ranging from 5.5 to 15 a_0, which was contracted to 15 basis functions with the aid of a method[35] that uses a radial cut of the potential through its minimum plus a term linear in R with a slope of 220 cm$^{-1}/a_0$ that resulted from minimizing the energies of the VRT levels. The angular expansion of the potential used in this method included values of L_A and L_B ranging from 0 to 10, L from 0 to 20, and K_A and K_B from -10 to 10 in steps of 2, because of the symmetry. The total number of terms in this expansion was 43010. The energies of the VRT levels calculated with this second method agree to about 0.1 cm^{-1} with those calculated with the pseudospectral method.

Both sets of calculations were performed for total angular momenta $J = 0$, 1, and 2. The molecular masses are 33.9877207542 u, and the rotational constants of H_2S taken from the literature[36] are $A = 10.3600$ cm^{-1}, $B = 9.0185$ cm^{-1}, and $C = 4.7306$ cm^{-1}.

In the theoretical spectrum calculated with the Lanczos-based method we included all allowed ($\Delta J = 0$ and ± 1) transitions, for initial states of all symmetries with total angular momenta $J = 0$, 1, 2, and 3. Since we noticed that the energies of the VRT levels were already converged to about 0.1 cm^{-1} with a basis containing maximum monomer angular momenta of 8, we used this somewhat smaller analytic angular basis and a correspondingly smaller grid

basis. Spectra generated for each symmetry by analytically calculating the transition dipole moments between the initial and final states were obtained from the VRT wave functions for $J = 0$, 1, and 2. The expansion of the dipole function used in the latter method contained 2541 terms with maximum angular momenta $L_{\rm A}$ and $L_{\rm B}$ of 4 and components $k = -1, 0, 1$, see Eqs. (35) to (38) in Ref. 26. So the latter spectra are less complete and less accurate, but the transition frequencies and line strengths calculated by this analytical method are helpful in assigning the peaks in the spectrum generated with the Lanczos-based method.

Results

The total angular momentum J is a good quantum number and the lower bound VRT levels calculated for $J = 0$, 1, and 2 with the pseudospectral method and the basis with $j_{\rm A}^{\rm max} = j_{\rm B}^{\rm max} = 10$ are listed in Tables 3, 4, and 5 for pH_2S-pH_2S, oH_2S-oH_2S, and oH_2S-pH_2S, respectively. The tunneling levels are displayed graphically in Fig. 1 of the main paper and the levels including intermolecular vibrations in Fig. 2. The energy levels can also be characterized with an approximate quantum number K, which is the projection of the total angular momentum J on the intermolecular axis $\boldsymbol{R}$. We use the absolute value of K, because the VRT wave functions are even/odd combinations of functions with $+K$ and $-K$. The energies in the tables are the intermolecular vibrational and tunneling levels, with dimer rotational quantum numbers $J \geq K$. From the differences between the energies with the same K and $J = 0$, 1, 2 one can derive the dimer end-over-end rotational constant $(B + C)/2$. If the dimer were a rigid rotor the differences between the energies for different K would yield the rotational constant A, but actually it is quite floppy and K affects its internal motions.

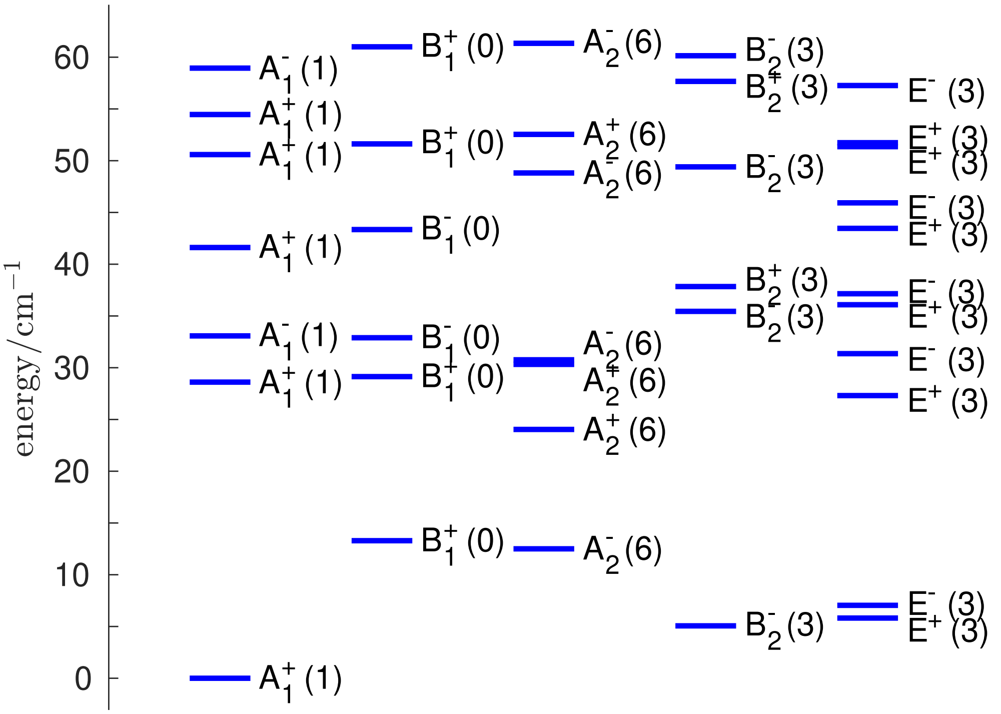

Suppl. Fig. 2: Vibrational energy levels of H_2S-H_2S for $J = K = 0$ up to 65 cm^{-1}. The nuclear spin weights are given in parentheses.

Suppl. Table 3: pH_2S-pH_2S levels (cm^{-1}), zero-point energy 241.0808 cm^{-1}. The approximate quantum number K is the projection of the total angular momentum J on the intermolecular axis.

K	irrep	$J = 0$	irrep	$J = 1$	irrep	$J = 2$
0	A_1^+	0.0000	B_1^-	0.1186	A_1^+	0.3558
1			B_1^-	4.6514	A_1^+	4.8880
2					A_1^+	17.1833
0	A_1^+	28.6081	B_1^-	28.7286	A_1^+	28.9695
1			B_1^-	31.5158	A_1^+	31.7588
1			B_1^-	37.8338	A_1^+	38.0700
2					A_1^+	39.8012
0	A_1^+	41.6115	B_1^-	41.7242	A_1^+	41.9495
1			B_1^-	44.3839	A_1^+	44.6240
0	A_1^+	50.5904	B_1^-	50.7104		
0	A_1^+	54.4610	B_1^-	54.5774		
1			B_1^+	17.8699	A_1^-	18.0997
2					A_1^-	28.4842
0	A_1^-	33.0743	B_1^+	33.1841	A_1^-	33.4037
1			B_1^+	34.3704	A_1^-	34.6145
1			B_1^+	36.6494	A_1^-	36.8936
2					A_1^-	45.7190
2					A_1^-	50.5066
0	A_1^-	58.9432	B_1^+	59.0610	A_1^-	59.2975
1			B_1^+	59.5043	A_1^-	59.7389
1			B_1^+	65.2229		
0	B_1^+	13.2876	A_1^-	13.4020	B_1^+	13.6309
1			A_1^-	17.8698	B_1^+	18.0993
2					B_1^+	28.4842
0	B_1^+	29.1396	A_1^-	29.2573	B_1^+	29.4928
1			A_1^-	34.3690	B_1^+	34.6107
1			A_1^-	36.6471	B_1^+	36.8866
2					B_1^+	45.7190
2					B_1^+	50.5066
0	B_1^+	51.6223	A_1^-	51.7373		
1			A_1^-	59.5019		
0	B_1^+	61.0019	A_1^-	61.1154		
1			A_1^+	4.6519	B_1^-	4.8898
2					B_1^-	17.1833
1			A_1^+	31.5145	B_1^-	31.7550
0	B_1^-	32.8954	A_1^+	33.0153	B_1^-	33.2552
1			A_1^+	37.8344	B_1^-	38.0716
2					B_1^-	39.8012
0	B_1^-	43.3462	A_1^+	43.4600	B_1^-	43.6875
1			A_1^+	44.3813	B_1^-	44.6161
2					B_1^-	50.1228
1			A_1^+	53.2730	B_1^-	53.5095
1			A_1^+	59.2228		
1			A_1^+	61.7977		
1			A_1^+	63.7727		

Suppl. Table 4: oH_2S-oH_2S levels (cm^{-1}), zero-point energy 246.1423 cm^{-1}. The approximate quantum number K is the projection of the total angular momentum J on the intermolecular axis.

K	irrep	$J = 0$	irrep	$J = 1$	irrep	$J = 2$
0	B_2^-	0.0000	A_2^+	0.1174	B_2^-	0.3521
1			A_2^+	3.7701	B_2^-	4.0052
2					B_2^-	14.8785
1			A_2^+	23.3375	B_2^-	23.5843
1			A_2^+	27.8145	B_2^-	28.0439
0	B_2^-	30.3832	A_2^+	30.5046	B_2^-	30.7475
2					B_2^-	32.7148
1			A_2^+	33.8786	B_2^-	34.1161
2					B_2^-	39.4883
0	B_2^-	44.3419	A_2^+	44.4560	B_2^-	44.6842
1			A_2^+	52.7260		
0	B_2^-	55.0775				
1			A_2^-	10.4803	B_2^+	10.7132
2					B_2^+	19.8862
1			A_2^-	31.1450	B_2^+	31.3859
0	B_2^+	32.7783	A_2^-	32.8924	B_2^+	33.1207
1			A_2^-	37.0463	B_2^+	37.2731
2					B_2^+	44.0039
2					B_2^+	48.0832
1			A_2^-	51.5577		
0	B_2^+	52.6022	A_2^-	52.7200		
1			A_2^-	54.8261		
0	A_2^-	7.4418	B_2^+	7.5580	A_2^-	7.7905
1			B_2^+	10.4798	A_2^-	10.7119
2					A_2^-	19.8862
0	A_2^-	25.7031	B_2^+	25.8237	A_2^-	26.0647
1			B_2^+	31.1452	A_2^-	31.3866
1			B_2^+	37.0463	A_2^-	37.2731
0	A_2^-	43.7533	B_2^+	43.8675	A_2^-	44.0963
2					A_2^-	48.0833
1			B_2^+	51.5585		
1			B_2^+	54.8294		
0	A_2^-	56.2797	B_2^+	56.3969		
1			B_2^-	3.7694	A_2^+	4.0031
2					A_2^+	14.8785
0	A_2^+	18.9753	B_2^-	19.0987	A_2^+	19.3456
1			B_2^-	23.3350	A_2^+	23.5770
0	A_2^+	25.2618	B_2^-	25.3798	A_2^+	25.6159
1			B_2^-	27.8159	A_2^+	28.0479
2					A_2^+	32.7148
1			B_2^-	33.8783	A_2^+	34.1153
2					A_2^+	39.4880
2					A_2^+	46.8192
0	A_2^+	47.4817	B_2^-	47.6035		
1			B_2^-	52.7266		

Suppl. Table 5: oH_2S-pH_2S levels (cm^{-1}), zero-point energy 246.8949 cm^{-1}. The approximate quantum number K is the projection of the total angular momentum J on the intermolecular axis.

K	irrep	$J = 0$	irrep	$J = 1$	irrep	$J = 2$
0	E^+	0.0000	E^-	0.1176	E^+	0.3529
1			E^-	3.9412	E^+	4.1767
1			E^-	4.5518	E^+	4.7872
2					E^+	15.4766
2					E^+	15.7388
0	E^+	21.4915	E^-	21.6119	E^+	21.8525
1			E^-	25.5436	E^+	25.7832
0	E^+	30.2709	E^-	30.3835	E^+	30.6095
1			E^-	32.7347		
0	E^+	37.6491				
0	E^+	45.5425				
0	E^+	45.9239				
0	E^-	1.2297	E^+	1.3466	E^-	1.5804
1			E^+	3.9419	E^-	4.1790
1			E^+	4.5512	E^-	4.7853
2					E^-	15.4766
2					E^-	15.7388
1			E^+	25.5279	E^-	25.7434
0	E^-	25.5440	E^+	25.6787	E^-	25.9414
1			E^+	27.6479	E^-	27.8828
0	E^-	31.3337	E^+	31.4448		
1			E^+	32.7369		
1			E^+	33.7259		
0	E^-	40.1138				
0	E^-	51.4470				

We can identify the tunneling processes in H_2S-H_2S that give rise to the level splittings in Fig. 1 of the main paper by looking at the VRT wave functions, which we plotted for $J = 0$. Acceptor switch tunneling interchanges the H atoms in the acceptor by the permutation P_{12}. The ground state A_1^+ wave function is symmetric with respect to P_{12}. The B_2^- wave function is antisymmetric, so the lowest B_2^- state is the acceptor switch tunneling excited state. This is clearly illustrated in Fig. 3, which shows both wave functions as functions of the coordinates β_{acceptor} and α in the region of two relevant minima of the potential. The coordinate β_{acceptor} is the angle between the acceptor C_2 axis and the intermolecular axis

$\boldsymbol{R}$ and α is the dihedral or torsional angle. The A_1^+ wave function is indeed symmetric, while the B_2^- wave function has a nodal plane in between the two absolute maxima in the wave function located at the minima in the potential. This implies that the acceptor switch tunneling splitting is 5.06 cm^{-1}, which is considerably smaller than the splitting of about 11 cm^{-1} for H_2O-H_2O. This is probably related to the acceptor plane being nearly perpendicular to the hydrogen bond in H_2S-H_2S, which implies that the acceptor tunneling pathway is longer than in H_2O-H_2O. This splitting cannot be measured, however, because the A_1^+ state belongs to pH_2S-pH_2 and the B_2^- state to oH_2S-oH_2 and the transition between these states is strictly forbidden.

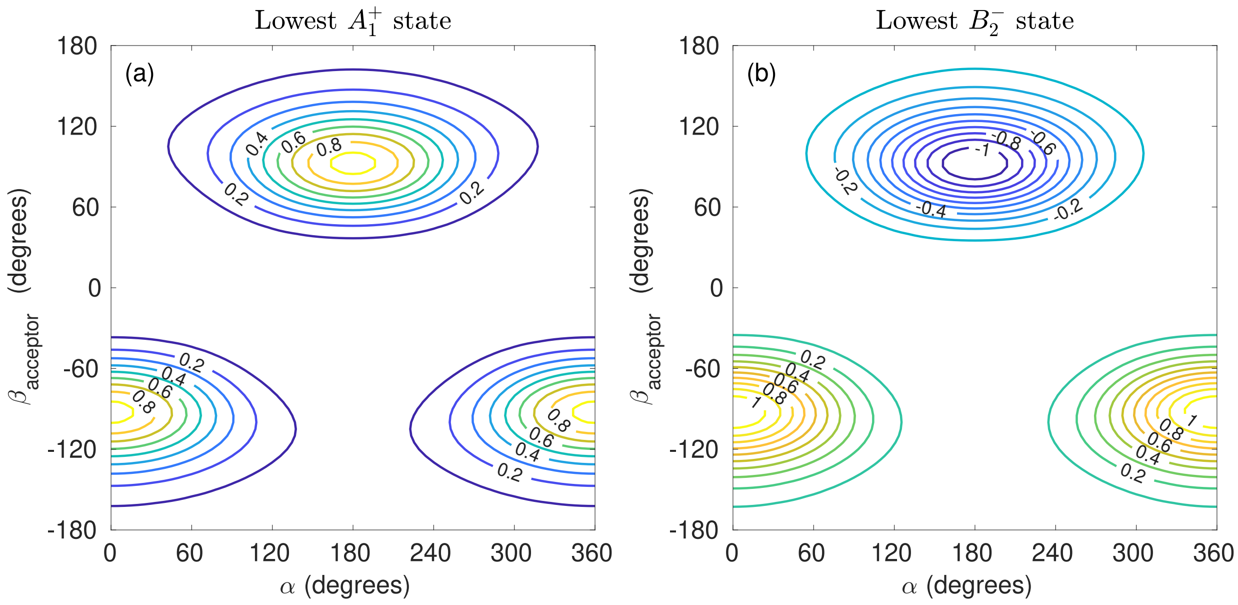

Suppl. Fig. 3: Wave functions of the acceptor switch tunneling states for $J = 0$: **(a)** lower state of A_1^+ symmetry and **(b)** upper state of B_2^- symmetry. Coordinates not shown are kept at their equilibrium values.

The donor-acceptor interchange process may best be modeled with the coordinates $\chi_{\rm D}$ and $\chi_{\rm A}$ that cover the region including two relevant donor-acceptor and acceptor-donor minima in the potential.[37] These coordinates are defined by Eqs. (11) and (12) in Ref. 37. The angle $\chi_{\rm D}$ rotates the donor molecule about an axis $\boldsymbol{n}_{\rm D}$ from $\chi_{\rm D} = 0^\circ$ to the acceptor orientation at $\chi_{\rm D} = 95^\circ$ and the angle $\chi_{\rm A}$ rotates the acceptor about an axis $\boldsymbol{n}_{\rm A}$ from $\chi_{\rm A} = 0^\circ$ to the donor orientation at $\chi_{\rm A} = 95^\circ$. The tunneling permutation in this case is P_{AB} and

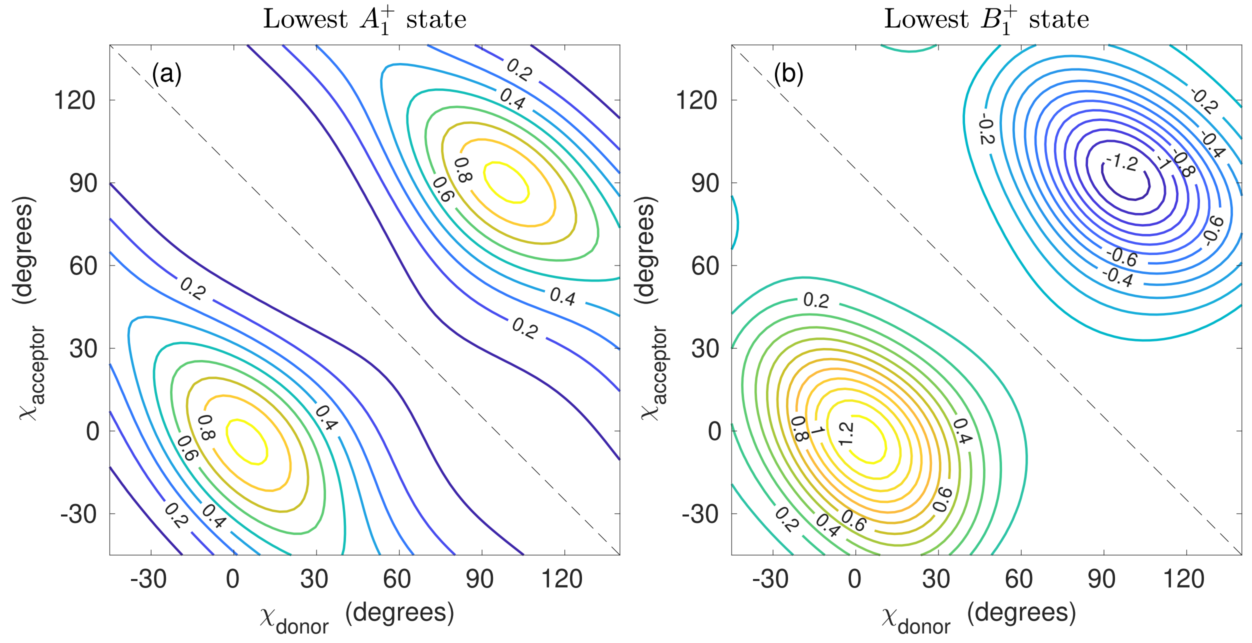

Suppl. Fig. 4: Wave functions of the interchange tunneling states for $J = 0$: **(a)** lower state of A_1^+ symmetry and **(b)** upper state of B_1^+ symmetry. The angles χ_{donor} and χ_{acceptor} are defined in the text and in Ref. 37; $\chi_{\text{donor}} = \chi_{\text{acceptor}} = 0°$ is the donor-acceptor equilibrium structure and $\chi_{\text{donor}} = \chi_{\text{acceptor}} = 95°$ the acceptor-donor structure. The distance R is kept at its equilibrium value of $7.72\,a_0$.

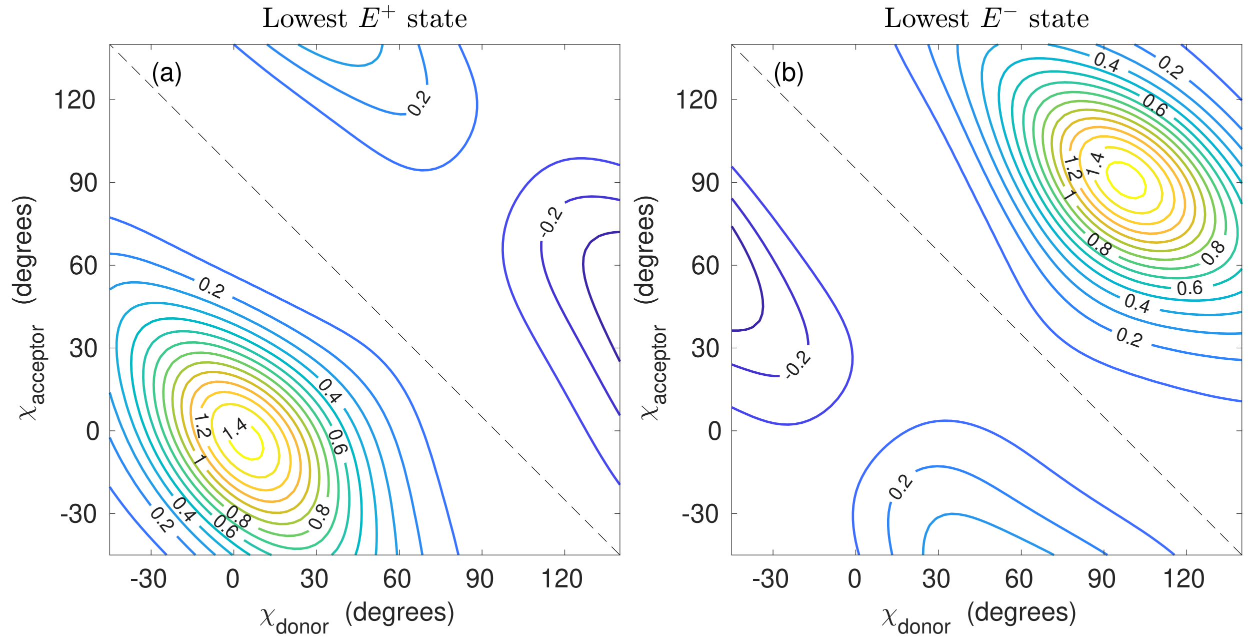

Suppl. Fig. 5: Wave functions of the lowest E^+ state **(a)** and E^- state **(b)** for $J = 0$. The coordinates are the same as in Fig. 4.

the tunneling excited state has B_1^+ symmetry. The wave functions are shown in Fig. 4. The A_1^+ state is symmetric again and the B_1^+ state has a nodal plane between the two maxima in the absolute wave function that occur at the minima in the potential. So the interchange tunneling splitting is 13.29 cm^{-1}.

Fig. 5 shows the lowest E^+ and E^- states for the same coordinates as used in Fig. 4. This figure clearly shows that the $E^\pm$ states are not symmetric with respect to interchange, which is because they correspond to the mixed ortho-para dimer. The lowest E^+ state in which oH_2S is the donor and pH_2S the acceptor is lower in energy by 1.23 cm^{-1} than the E^- state in which it is the other way around. This is because the monomer rigid rotor functions in the angular basis have odd $k_a + k_c$ for oH_2S starting with $j_{k_a k_c} = 1_{01}$ and even $k_a + k_c$ for pH_2S starting with 0_{00}. For the donor the internal rotation is more strongly quenched than for the acceptor, which is more easily realized with the oH_2S basis that starts with the 1_{01} function than with the pH_2S basis that starts with the isotropic 0_{00} function. Because of this asymmetry the $E^\pm$ states are the only states that have a non-zero dipole moment. The $A_{1,2}^\pm$ and $B_{1,2}^\pm$ states are symmetric or antisymmetric under P_{AB}, they all have equal weights for donor-acceptor and acceptor-donor structures, and their dipole moment averages to zero. Therefore, the microwave spectrum[38] of H_2S-H_2S probes only the E^+ and E^- states.

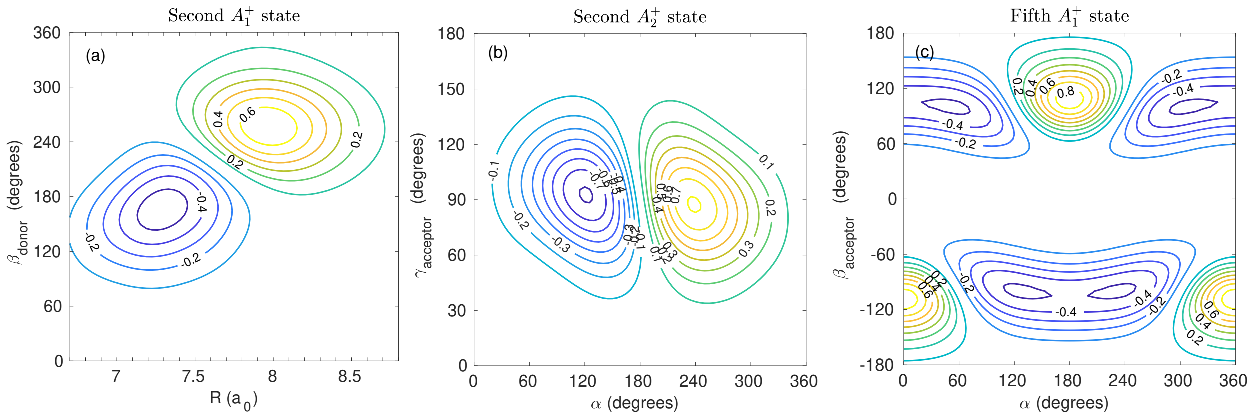

Suppl. Fig. 6: Wave functions of some vibrationally excited states for $J = 0$: **(a)** mixed stretch and donor bend excited state of A_1^+ symmetry at energy 28.6 cm^{-1}, **(b)** mixed torsionally and acceptor twist excited state of A_2^+ symmetry at 25.3 cm^{-1} above the B_2^- ground state of oH_2S-oH_2S, and **(c)** mixed torsion and acceptor wag excited state of A_1^+ symmetry at 54.5 cm^{-1}.

Finally Fig. 6 shows the wave functions of some vibrational states excited in the intermolecular coordinates. The angle β_{donor} is the angle between the donor C_2 axis and the intermolecular axis $\boldsymbol{R}$ and γ_{acceptor} rotates the acceptor monomer about its C_2 axis. The nodal planes in these pictures are not horizontal or vertical, which illustrates that different intermolecular modes are mixed.

Suppl. Note 3 VSCF/VCI calculations

VSCF/VCI calculations of the four SH stretch modes in the H_2S dimer were performed on the 12D PES with the code MULTIMODE, Version 5.1.4.[39–41] For all the calculations, a four-mode representation of the potential in mass-scaled normal coordinates and a three-mode representation of the effective inverse moment of inertia for the vibrational angular momentum terms in the exact Watson Hamiltonian are used.[42] The formalism is based on CI from the virtual space of the ground vibrational state VSCF Hamiltonian. Here we explore reduced-mode coupling models, i.e., 6 intramolecular modes, where this set of modes starts with the highest frequency and proceeds in decreasing frequency. In this calculation, the maximum mode combination excitations are 10, 10, 10, 8, which means that single through triple excitations extend to a maximum sum of quanta of 10, and that for quadruple excitations the maximum is 8. This excitation space leads to the CI matrix size of 4186 and we compute 20 CI vibrational states up to the energy of 4000 cm^{-1}.

Calculations were done at each of the three minima in the potential with small differences of 10-20 cm^{-1} for the SH-stretch fundamentals among them. The IR spectra for each of these minima obtained with MULTIMODE using the full-dimensional DMS are shown in Fig. 7.

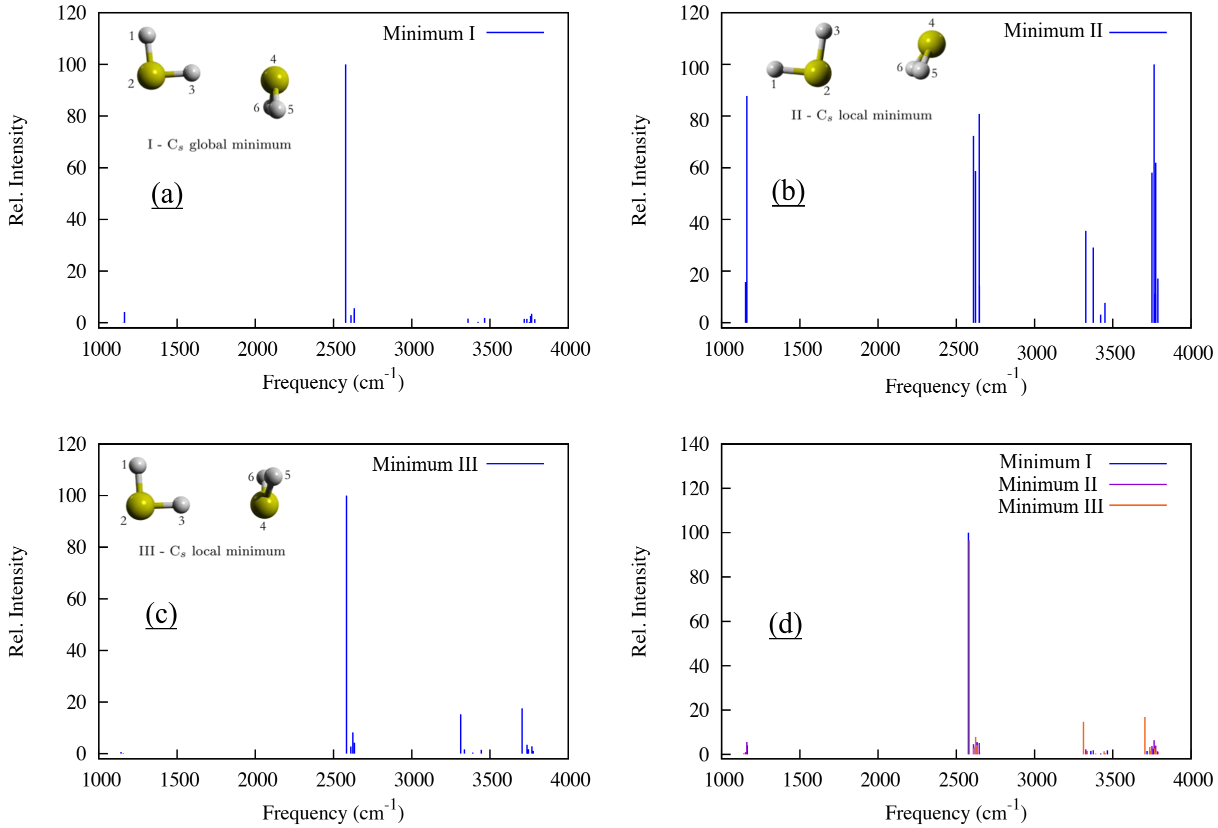

Suppl. Fig. 7: Anharmonic vibrational spectra at the three minima of the H_2S dimer potential: **(a)** Minimum I, **(b)** Minimum II, **(c)** Minimum III, **(d)** combined spectrum.

Suppl. Note 4 Diffusion Monte Carlo calculations

The 12D PES is also applied to compute the rigorous quantum zero-point energy (ZPE) of H_2S dimer using unbiased DMC calculations. The basic goal of the diffusion Monte Carlo (DMC) method is to solve the time-dependent Schrödinger equation in imaginary time.[43–45] This is done by simulating a random walk of many replicas, also called "walkers", of the molecule, using a birth/death process. At each step, a random displacement in each degree of freedom is assigned to each walker, and this walker may remain alive (and may give birth to a new walker) or be killed by comparing its potential energy, E_i, with reference energy, E_r. For the ground state, the probability of birth or death is given as:

$$P_{\mathrm{birth}} = \exp\left[-(E_i - E_r)\Delta\tau\right] - 1 \ (E_i < E_r) \tag{4}$$

$$P_{\mathrm{death}} = 1 - \exp\left[-(E_i - E_r)\Delta\tau\right] \ (E_i > E_r), \tag{5}$$

where $\Delta\tau$ is the step size in imaginary time. After removing all dead walkers, the reference energy is updated using the equation

$$E_r(\tau) = \langle V(\tau)\rangle - \alpha\frac{N(\tau) - N(0)}{N(0)}, \tag{6}$$

where τ is the imaginary time; $\langle V(\tau)\rangle$ is the average potential over all the walkers that are alive; $N(\tau)$ is the number of live walkers at time τ. The parameter α controls the fluctuations in the number of walkers and the reference energy. Finally, the average of the reference energy over the imaginary time gives an estimate of the ZPE.

In this study, each DMC trajectory is propagated for 40,000 time steps with a stepsize of 5.0 a.u.; 30,000 steps are used to equilibrate the walkers, and the reference energies in the remaining 10,000 steps are used to compute the ZPE. For each of the three equilibrium geometries, 10 DMC simulations (or trajectories) were carried out with 30,000 random walkers, and the final ZPE is the average of the 10 simulations. The statistical uncertainty of

the zero-point energy is defined as the standard deviation of DMC energies over the total number of simulations. It is written as

$$\Delta E = \sqrt{\frac{1}{10}\sum_{i=1}^{10}(E_i - \bar{E})^2}, \tag{7}$$

where $\bar{E}$ is the average energy over the 10 simulations.

Histograms of the DMC wavefunction for the H_2S dimer are shown in Fig. 8. Analogous histograms are shown for the H_2O dimer in Fig. 9. These were obtained using the dimer component of the q-AQUA-pol potential.[46] Even a cursory glance at these sets of histograms shows signficantly more delocalization of the wavefunction “floppiness" for the H_2S dimer compared to the H_2O dimer.

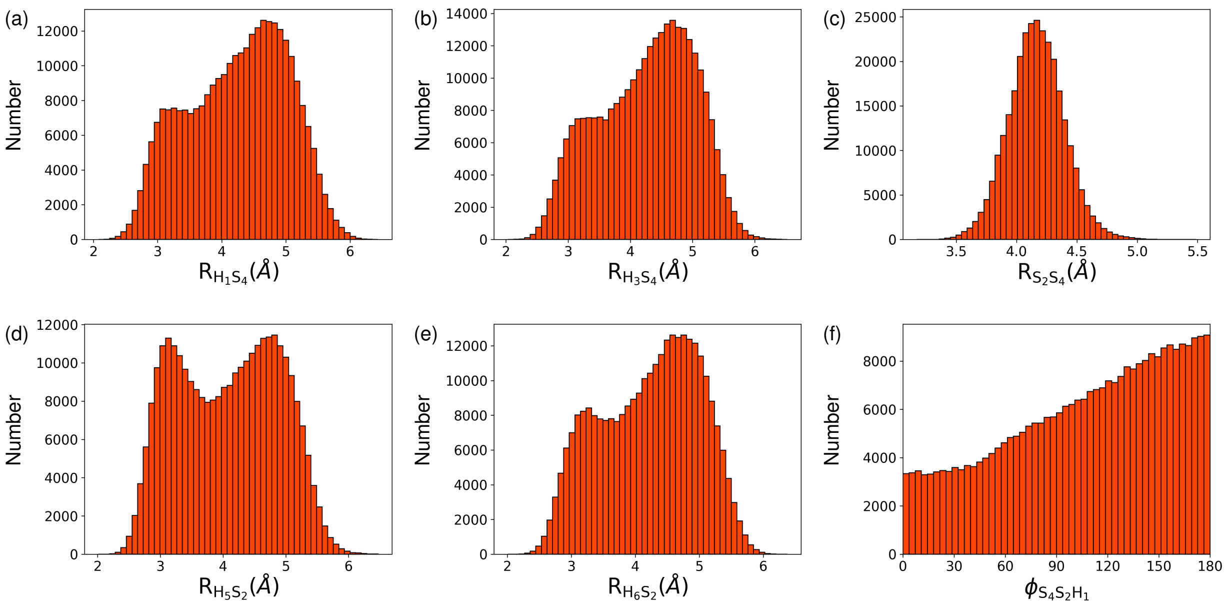

Suppl. Fig. 8: Distribution of walkers for H_2S dimer (Minimum I) as a function of the (a-b,d-e) SH internuclear distances, (c) the SS internuclear distance, and (f) the SSH dihedral angle.

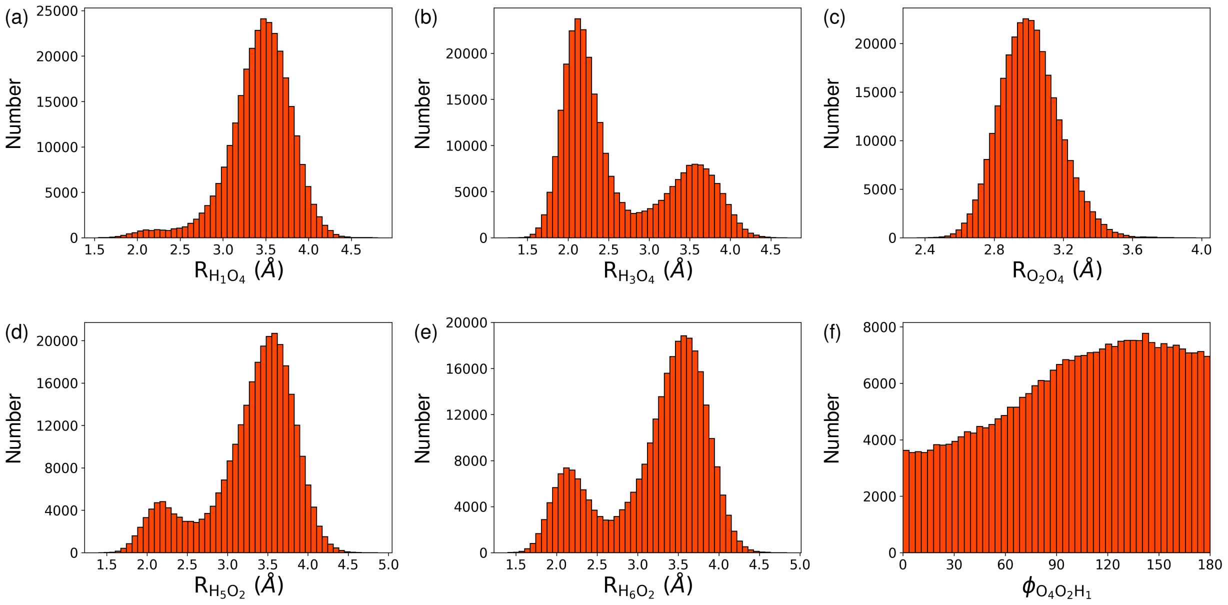

Suppl. Fig. 9: Distribution of walkers for H_2O dimer as a function of the (a-b,d-e) OH internuclear distances, (c) the OO internuclear distance, and (f) the OOH dihedral angle.

Suppl. Note 5 SAPT Analysis

The structures of the three H_2S dimer minima identified in Ref. 7 have been reoptimized using the CCSD(T) coupled-cluster method[9–11] and the aug-cc-pV5Z basis set[16,18] (denoted a5Z) in `CFOUR`[47,48] which was followed by harmonic vibrational frequency computations obtained from finite differences of analytic gradients. An analysis based on symmetry-adapted perturbation theory (SAPT)[49–51] was carried out on each optimized structure using higher-order SAPT2+3(CCD) computations to treat dispersion with a method based on coupled-cluster doubles that can offer improvements in challenging cases such as the PCCP dimer.[52,53] These SAPT2+3(CCD) computations were carried out with the a5Z basis set using the efficient implementation in the `PSI4`[54,55] quantum chemistry software package that employs natural orbital truncation.[56] Additional CCSD(T) single-point energies were computed using `Molpro`[22,23] on the aforementioned optimized geometries with the aug-cc-pVQZ and aug-cc-pV6Z basis sets in order to calculate relative electronic energies near the complete basis set (CBS) limit, see Table 6. The Hartree-Fock (HF) energy at the CBS limit was obtained

by using all three basis sets from quadruple-ζ to sextuple-ζ in an algebraic form[57–59] of the three-parameter exponential function proposed by Feller.[60]

$$E_{\mathrm{HF}}^{\mathrm{CBS}} = E_{\mathrm{HF}}^{\mathrm{a6Z}} - \frac{(E_{\mathrm{HF}}^{\mathrm{a6Z}} - E_{\mathrm{HF}}^{\mathrm{a5Z}})^2}{E_{\mathrm{HF}}^{\mathrm{aQZ}} - 2E_{\mathrm{HF}}^{\mathrm{a5Z}} + E_{\mathrm{HF}}^{\mathrm{a6Z}}} \tag{8}$$

Similarly, the electronic correlation energy at the CBS limit was calculated using an algebraic expression[57–59] for the two-parameter inverse cubic function described by Helgaker and co-workers[61] with the pentuple-ζ and sextuple-ζ basis sets.

$$E_{\mathrm{c}}^{\mathrm{CBS}} = \frac{216E_{\mathrm{c}}^{\mathrm{a6Z}} - 125E_{\mathrm{c}}^{\mathrm{a5Z}}}{91} \tag{9}$$

The default frozen-core approximation was used in all computations (i.e., freezing the 1s-, 2s- and 2p-like orbitals of S in the electron correlation procedures).

The SAPT2+3(CCD) computations reveal that dispersion and electrostatics have nearly equal contributions to the interaction energies for all three minima of the H_2S dimer. These computations with the a5Z basis set yielded total interaction energies of −7.5, −7.7 and −7.1 kJ mol^{-1} for the CCSD(T)/a5Z optimized geometries of Minimum I, II, and III, respectively, with nearly equal attractive contributions from electrostatics (−9.4, −8.0 and −9.0 kJ mol^{-1}) and dispersion (−7.9, −9.2 and −7.8 kJ mol^{-1}). For comparison, the same analysis on the water dimer global minimum reveals an interaction energy of −21.7 kJ mol^{-1} and a much larger contribution from electrostatics (−33.6 kJ mol^{-1}) relative to dispersion (−10.7 kJ mol^{-1}). These results are reported in Table 7 along with the contributions from induction and exchange repulsion. The geometries are given in Table 8.

Suppl. Table 6: CCSD(T) relative electronic energies (in cm^{-1}) of the three H_2S dimer minima and two isolated monomers (i.e., the dissociation energy (D_e) of Minimum I) computed using the CCSD(T)/a5Z optimized structures.

Basis Set	Minimum I	Minimum II	Minimum III	D_e
aQZ	0	31	30	617
a5Z	0	23	28	602
a6Z	0	19	29	593
CBS[a]	0	14	30	583

[a]Calculated using Eqs. (8) and (9)

Suppl. Table 7: Components of and the total interaction energy (in kJ mol^{-1}) from SAPT2+3(CCD) computations with the a5Z basis on the CCSD(T)/a5Z optimized structures of Minimum I, II and III of the H_2S dimer as well as the global minimum of the H_2O dimer.

Component	Minimum I	Minimum II	Minimum III	$(H_2O)_2$
Electrostatics	−9.4	−8.0	−9.0	−33.6
Exchange Repulsion	+13.2	+11.1	+13.1	+32.6
Induction	−3.4	−1.6	−3.4	−10.1
Dispersion	−7.9	−9.2	−7.8	−10.7
Total	−7.5	−7.7	−7.1	−21.7

Suppl. Table 8: Cartesian coordinates (in a.u.) of the CCSD(T)/a5Z optimized monomer and dimer structures.

Monomer	X	Y	Z
S	0.000000	0.000000	0.103828
H	0.000000	-1.824618	-1.646904
H	0.000000	1.824618	-1.646904
Minimum I	X	Y	Z
S	-3.952350	0.085738	-0.000000
H	-4.384661	-2.405835	0.000000
H	-1.438365	-0.234533	0.000000
S	3.887495	-0.106076	0.000000
H	3.940236	1.642793	1.826941
H	3.940236	1.642793	-1.826941
Minimum II	X	Y	Z
S	3.460955	0.081536	-0.000000
H	2.428985	-2.225737	0.000000
H	5.822779	-0.824451	0.000000
S	-3.570405	-0.063079	-0.000000
H	-2.389797	1.232329	1.824649
H	-2.389797	1.232329	-1.824649
Minimum III	X	Y	Z
S	-3.975619	0.073582	0.000000
H	-4.093159	-2.452607	0.000000
H	-1.441396	0.068114	0.000000
S	3.888350	0.104057	0.000000
H	4.151538	-1.625441	-1.827055
H	4.151538	-1.625441	1.827055

Suppl. Note 6 Pick-up curves

We determined the cluster size by so-called pick-up curves. A detailed description of this method has been given elsewhere.[62–65] The pick-up of molecules by the helium droplets approximately follows Poisson statistics if the pick-up cross section (σ) and the length of the pick-up region (L) are considered constant. In this case, the number of molecules (k) picked up by the helium droplets can be directly correlated to the density (η) of those molecules in the pick-up region, which can be adjusted by changing the partial pressure inside the pick-up chamber.

$$P(\eta) = \frac{(\eta\sigma L)^k}{k!} e^{-(\eta\sigma L)} \tag{10}$$

Experimentally, the pick-up curves can be determined by setting the laser to the maximum of a signal and then recording the amplitude of the signal while changing the partial pressure in the pick-up chamber. In Fig. 10 this method was used to assign the signals in Fig. 3 of the paper to the H_2S dimer and trimer. The pick-up curves were recorded mass-selectively at $m/z = 34$ as in the IR spectrum in Fig. 3.

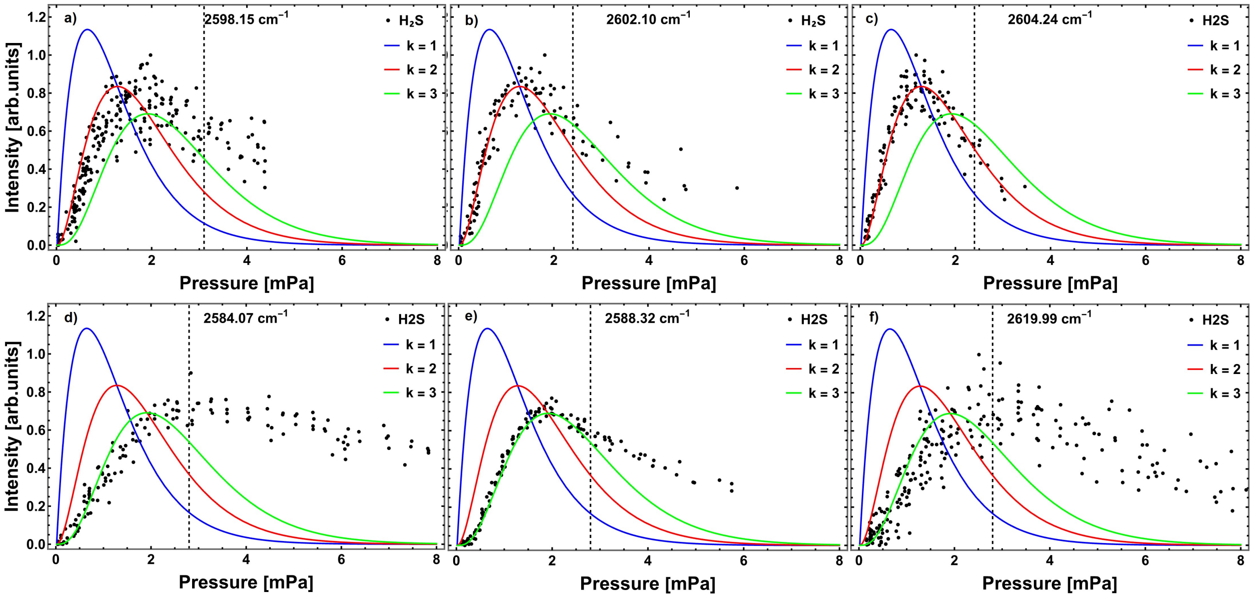

Suppl. Fig. 10: Pick-up curves of the IR bands reported in Fig. 3. They were recorded at a) 2598.15 cm^{-1}, b) 2602.10 cm^{-1}, and c) 2604.24 cm^{-1} and were assigned to the H_2S dimer, while the signals at d) 2584.07 cm^{-1}, e) 2588.32 cm^{-1}1, and f) 2619.99 cm^{-1} were assigned to the H_2S trimer. The pressure dependence of the signal intensity (black dots) was fitted to a Poisson distribution with $k = 1$, 2, and 3, respectively (with k referring to the number of H_2S molecules inside the helium droplets). The black dotted lines mark the cut-off pressure for the fits. All pick-up curves were recorded mass selectively at $m/z = 34$.

Supplementary references

(1) Nandi, A.; Qu, C.; Houston, P. L.; Conte, R.; Bowman, J. M. Δ-machine learning for potential energy surfaces: A PIP approach to bring a DFT-based PES to CCSD(T) level of theory. *J. Chem. Phys.* **2021**, *154*, 051102.

(2) Braams, B. J.; Bowman, J. M. Permutationally invariant potential energy surfaces in high dimensionality. *Int. Rev. Phys. Chem.* **2009**, *28*, 577–606.

(3) Bowman, J. M.; Braams, B. J.; Carter, S.; Chen, C.; Czakó, G.; Fu, B.; Huang, X.; Kamarchik, E.; Sharma, A. R.; Shepler, B. C.; Wang, Y.; Xie, Z. Ab-initio-based potential energy surfaces for complex molecules and molecular complexes. *J. Phys. Chem. Lett.* **2010**, *1*, 1866–1874.

(4) Xie, Z.; Bowman, J. M. Permutationally Invariant Polynomial Basis for Molecular Energy Surface Fitting via Monomial Symmetrization. *J. Chem. Theory Comput.* **2010**, *6*, 26–34.

(5) Bowman, J. M.; Czakó, G.; Fu, B. High-dimensional ab initio potential energy surfaces for reaction dynamics calculations. *Phys. Chem. Chem. Phys.* **2011**, *13*, 8094–8111.

(6) Qu, C.; Yu, Q.; Bowman, J. M. Permutationally invariant potential energy surfaces. *Annu. Rev. Phys. Chem.* **2018**, *69*, 6.1–6.25.

(7) Perkins, M. A.; Barlow, K. R.; Dreux, K. M.; Tschumper, G. S. Anchoring the hydrogen sulfide dimer potential energy surface to juxtapose $(H_2S)_2$ with $(H_2O)_2$. *J. Chem. Phys.* **2020**, *152*, 214306.

(8) Werner, H.-J.; Knowles, P. J.; Knizia, G.; Manby, F. R.; Schütz, M. MOLPRO, version 2015.1, a package of ab initio programs. 2015; see http://www.molpro.net.

(9) Bartlett, R. Many-Bondy Perturbation Theory and Coupled Cluster Theory for Electron Correlation in Molecules. *Ann. Rev. Phys. Chem.* **1981**, *32*, 359–401.

(10) Purvis, G.; Bartlett, R. A full coupled-cluster singles and doubles model: The inclusion of disconnected triples. *J. Chem. Phys.* **1982**, *76*, 1910–1918.

(11) Raghavachari, K.; Trucks, G. W.; Pople, J. A.; Head-Gordon, M. A fifth-order perturbation comparison of electron correlation. *Chem. Phys. Lett.* **1989**, *157*, 479–483.

(12) Adler, T. B.; Knizia, G.; Werner, H.-J. A simple and efficient CCSD(T)-F12 approximation. *J. Chem. Phys.* **2007**, *127*, 221106.

(13) Knizia, G.; Adler, T. B.; Werner, H.-J. Simplified CCSD(T)-F12 methods: Theory and benchmarks. *J. Chem. Phys.* **2009**, *130*, 054104.

(14) Hättig, C.; Tew, D. P.; Köhn, A. Communications: Accurate and efficient approximations to explicitly correlated coupled-cluster singles and doubles, CCSD-F12. *J. Chem. Phys.* **2010**, *132*, 231102.

(15) Werner, H.-J.; Knizia, G.; Manby, F. R. Explicitly correlated coupled cluster methods with pair-specific geminals. *Mol. Phys.* **2011**, *109*, 407–417.

(16) Dunning, Jr., T. H. Gaussian Basis Sets for Use in Correlated Molecular Calculations. I. the Atoms Boron Through Neon and Hydrogen. *J. Chem. Phys.* **1989**, *90*, 1007–1023.

(17) Peterson, K. A.; Adler, T. B.; Werner, H.-J. Systematically convergent basis sets for explicitly correlated wavefunctions: The atoms H, He, B–Ne, and Al–Ar. *J. Chem. Phys.* **2008**, *128*, 084102.

(18) Woon, D. E.; Dunning Jr., T. H. Gaussian basis sets for use in correlated molecular calculations. III. The atoms aluminum through argon. *J. Chem. Phys.* **1993**, *98*, 1358–1371.

(19) Weigend, F. A fully direct RI-HF algorithm: Implementation, optimised auxiliary basis sets, demonstration of accuracy and efficiency. *Phys. Chem. Chem. Phys.* **2002**, *4*, 4285–4291.

(20) Weigend, F.; Köhn, A.; Hättig, C. Efficient use of the correlation consistent basis sets in resolution of the identity MP2 calculations. *J. Chem. Phys.* **2002**, *116*, 3175–3183.

(21) Yousaf, K. E.; Peterson, K. A. Optimized auxiliary basis sets for explicitly correlated methods. *J. Chem. Phys.* **2008**, *129*, 184108.

(22) Werner, H. J.; Knowles, P. J.; Knizia, G.; Manby, F. R.; Scchutz, M. *WIREs Comput. Mol. Sci.* **2012**, *2*, 242–253.

(23) Werner, H.-J. et al. MOLPRO, version 2019.2, a package of ab initio programs. 2018; see https://www.molpro.net.

(24) Huang, X.; Braams, B. J.; Bowman, J. M. Ab initio potential energy and dipole moment surfaces for $H_5O_2^+$. *J. Chem. Phys.* **2005**, *122*, 044308.

(25) Brocks, G.; van der Avoird, A.; Sutcliffe, B. T.; Tennyson, J. Quantum dynamics of non-rigid systems comprising two polyatomic fragments. *Mol. Phys.* **1983**, *50*, 1025.

(26) van der Avoird, A.; Wormer, P. E. S.; Moszynski, R. From intermolecular potentials to the spectra of Van der Waals molecules, and vice versa. *Chem. Rev.* **1994**, *94*, 1931–1974.

(27) Leforestier, C.; Braly, L. B.; Liu, K.; Elrod, M. J.; Saykally, R. J. Fully coupled six-dimensional calculations of the water dimer vibration-rotation-tunneling states with a split Wigner pseudo spectral approach. *J. Chem. Phys.* **1997**, *106*, 8527.

(28) Lanczos, C. An iteration method for the solution of the eigenvalue problem of linear differential and integral operators. *J. Res. Natl. Bur. Stand.* **1950**, *45*, 255.

(29) Davidson, E. R. The iterative calculation of a few of the lowest eigenvalues and corresponding eigenvectors of large real-symmetric matrices. *J. Comput. Phys.* **1975**, *17*, 87.

(30) Groenenboom, G. C.; Wormer, P. E. S.; van der Avoird, A.; Mas, E. M.; Bukowski, R.; Szalewicz, K. Water pair potential of near spectroscopic accuracy: II. Vibration-rotation-tunneling levels of the water dimer. *J. Chem. Phys.* **2000**, *113*, 6702–6715.

(31) Wyatt, R. E.; Scott, D. S. *North-Holland Mathematics Studies 127C*; North Holland, 1986; pp 67–79.

(32) Wyatt, R. E. The recursive residue generation method. *Adv. Chem. Phys.* **1989**, *73*, 231.

(33) Ziemkiewicz, M. P.; Pluetzer, C.; Nesbitt, D. J.; Scribano, Y.; Faure, A.; van der Avoird, A. Overtone vibrational spectroscopy in H_2-H_2O complexes: A combined high level theoretical *ab initio*, dynamical and experimental study. *J. Chem. Phys.* **2012**, *137*, 084301.

(34) Bunker, P. R.; Jensen, P. *Molecular Symmetry and Spectroscopy*, 2nd ed.; NRC Research Press: Ottawa, 1998.

(35) van der Avoird, A.; Nesbitt, D. J. Rovibrational states of the H_2O-H_2 complex; an *ab initio* calculation. *J. Chem. Phys.* **2011**, *134*, 044314.

(36) Cazzoli, G.; Puzzarini, C. The rotational spectrum of hydrogen sulfide: The $H_2^{33}S$ and $H_2^{32}S$ isotopologues revisited. *J. Mol. Spectrosc.* **2014**, *298*, 31–37.

(37) Smit, M. J.; Groenenboom, G. C.; Wormer, P. E. S.; van der Avoird, A.; Bukowski, R.; Szalewicz, K. Vibrations, tunneling, and transition dipole moments in the water dimer. *J. Phys. Chem. A* **2001**, *105*, 6212.

(38) Das, A.; Mandal, P. K.; Lovas, F. J.; Medcraft, C.; Walker, N. R.; Arunan, E. The H_2S dimer is hydrogen-bonded: direct confirmation from microwave spectroscopy. *Angew. Chem. Int. Ed.* **2018**, *57*, 15199–15203.

(39) Bowman, J. M. Self-consistent Field Energies and Wavefunctions for Coupled Oscillators. *J. Chem. Phys.* **1978**, *68*, 608–610.

(40) Carter, S.; Culik, S. J.; Bowman, J. M. Vibrational Self-consistent Field method for Many-mode Systems: A New Approach and Application to the Vibrations of CO Adsorbed on Cu(100). *J. Chem. Phys.* **1997**, *107*, 10458–10469.

(41) Bowman, J. M.; Carter, S.; Huang, X. MULTIMODE: a Code to Calculate Rovibrational Energies of Polyatomic Molecules. *Int. Rev. Phys. Chem.* **2003**, *22*, 533–549.

(42) Watson, J. K. G. Simplification of the Molecular Vibration-Rotation Hamiltonian. *Mol. Phys.* **1968**, *15*, 479–490.

(43) Anderson, J. B. A random-walk simulation of the Schrödinger equation: H_3^+. *J. Chem. Phys.* **1975**, *63*, 1499–1503.

(44) Anderson, J. B. Quantum Chemistry by random walk. H ^2P, H_3^+ D_{3h} $^1A_1'$, H_2 $^3\Sigma_u^+$, H_4 $^1\Sigma_g^+$, Be ^1S. *J. Chem. Phys.* **1976**, *65*, 4121–4127.

(45) Kosztin, I.; Faber, B.; Schulten, K. Introduction to the diffusion Monte Carlo method. *Am. J. Phys.* **1996**, *64*, 633–644.

(46) Qu, C.; Yu, Q.; Houston, P. L.; Conte, R.; Nandi, A.; Bowman, J. M. Interfacing q-AQUA with a Polarizable Force Field: The Best of Both Worlds. *J. Chem. Theory. Comput.* **2023**, *19*, 3446–3459.

(47) Stanton, J. F.; Gauss, J.; Cheng, L.; Harding, M. E.; Matthews, D. A.; Szalay, P. G. CFOUR, Coupled-Cluster techniques for Computational Chemistry, a quantum-chemical program package. With contributions from A.A. Auer, R.J. Bartlett, U. Benedikt, C. Berger, D.E. Bernholdt, Y.J. Bomble, O. Christiansen, F. Engel, R. Faber, M. Heckert, O. Heun, M. Hilgenberg, C. Huber, T.-C. Jagau, D. Jonsson, J. Jusélius, T. Kirsch, K. Klein, W.J. Lauderdale, F. Lipparini, T. Metzroth, L.A.

Mück, D.P. O'Neill, D.R. Price, E. Prochnow, C. Puzzarini, K. Ruud, F. Schiffmann, W. Schwalbach, C. Simmons, S. Stopkowicz, A. Tajti, J. Vázquez, F. Wang, J.D. Watts and the integral packages MOLECULE (J. Almlöf and P.R. Taylor), PROPS (P.R. Taylor), ABACUS (T. Helgaker, H.J. Aa. Jensen, P. Jørgensen, and J. Olsen), and ECP routines by A. V. Mitin and C. van Wüllen. For the current version, see http://www.cfour.de (accessed 2024).

(48) Matthews, D. A.; Cheng, L.; Harding, M. E.; Lipparini, F.; Stopkowicz, S.; Jagau, T.-C.; Szalay, P. G.; Gauss, J.; Stanton, J. F. Coupled-cluster techniques for computational chemistry: The CFOUR program package. *J. Chem. Phys.* **2020**, *152*, 214108.

(49) Jeziorski, B.; Moszynski, R.; Szalewicz, K. Perturbation Theory Approach to Intermolecular Potential Energy Surfaces of van der Waals Complexes. *Chem. Rev.* **1994**, *94*, 1887–1930.

(50) Hohenstein, E. G.; Sherrill, C. D. Wavefunction methods for noncovalent interactions. *WIREs Comput. Mol. Sci.* **2012**, *2*, 304–326.

(51) Szalewicz, K. Symmetry-adapted perturbation theory of intermolecular forces. *WIREs Comput. Mol. Sci.* **2012**, *2*, 254–272.

(52) Hohenstein, E. G.; Sherrill, C. D. Density fitting of intramonomer correlation effects in symmetry-adapted perturbation theory. *J. Chem. Phys.* **2010**, *133*, 014101.

(53) Hohenstein, E. G.; Jaeger, H. M.; Carrell, E. J.; Tschumper, G. S.; Sherrill, C. D. Accurate Interaction Energies for Problematic Dispersion-Bound Complexes: Homogeneous Dimers of NCCN, P_2 and PCCP. *J. Chem. Theory Comput.* **2011**, *7*, 2842–2851.

(54) Turney, J. M. et al. PSI4: An open-source ab initio electronic structure program. *WIREs Comput. Mol. Sci.* **2012**, *2*, 556–565.

(55) Smith, D. G. A. et al. PSI4 1.4: Open-source software for high-throughput quantum chemistry. *J. Chem. Phys.* **2020**, *152*, 184108.

(56) Parrish, R. M.; Sherrill, C. D. Tractability gains in symmetry-adapted perturbation theory including coupled double excitations: CCD+ST(CCD) dispersion with natural orbital truncations. *J. Chem. Phys.* **2013**, *139*, 174102.

(57) Carrell, E. J.; Thorne, C. M.; Tschumper, G. S. Basis set dependence of higher-order correlation effects in π-type interactions. *J. Chem. Phys.* **2012**, *136*, 014103.

(58) Carrell, E. J.; Thorne, C. M.; Tschumper, G. S. Erratum: "Basis set dependence of higher-order correlation effects in π-type interactions" [J. Chem. Phys. 136, 014103 (2012)]. *J. Chem. Phys.* **2020**, *153*, 069901.

(59) Perkins, M. A.; Barlow, K. R.; Dreux, K. M.; Tschumper, G. S. Anchoring the hydrogen sulfide dimer potential energy surface to juxtapose $(H_2S)_2$ with $(H_2O)_2$. *J. Chem. Phys.* **2020**, *152*, 214306.

(60) Feller, D. The use of systematic sequences of wave functions for estimating the complete basis set, full configuration interaction limit in water. *J. Chem. Phys.* **1993**, *98*, 7059.

(61) Helgaker, T.; Klopper, W.; Koch, H.; Noga, J. Basis-set convergence of correlated calculations on water. *J. Chem. Phys.* **1997**, *106*, 9639.

(62) Lewerenz, M.; Schilling, B.; Toennies, J. P. Successive capture and coagulation of atoms and molecules to small clusters in large liquid helium clusters. *J. Chem. Phys.* **1995**, *102*, 8191–8207.

(63) Nauta, K.; Miller, R. E. The hydrogen fluoride dimer in liquid helium: A prototype system for studying solvent effects on hydrogen bonding. *J. Chem. Phys.* **2000**, *113*, 10158–10168.

(64) Stienkemeier, F.; Lehmann, K. K. Spectroscopy and dynamics in helium nanodroplets. *J. Phys. B: At. Mol. Opt. Phys.* **2006**, *39*, R127–R166.

(65) Toennies, J. P.; Vilesov, A. F. Superfluid helium droplets: a uniquely cold nanomatrix for molecules and molecular complexes. *Angew. Chem. Int. Ed.* **2004**, *43*, 2622–2648.
